# Survival Benefit of Neoadjuvant Chemotherapy with S-1 Plus Docetaxel for Locally Advanced Gastric Cancer: A Propensity Score-Matched Analysis

**DOI:** 10.1245/s10434-019-07299-7

**Published:** 2019-04-11

**Authors:** Masayuki Kano, Koichi Hayano, Hideki Hayashi, Naoyuki Hanari, Hisashi Gunji, Takeshi Toyozumi, Kentaro Murakami, Masaya Uesato, Satoshi Ota, Hisahiro Matsubara

**Affiliations:** Department of Frontier Surgery, Chiba University, Chiba, Japan

## Abstract

**Background:**

Postoperative docetaxel plus S-1 (DS) chemotherapy is expected to be the standard therapeutic strategy for pStage III gastric cancer based on the results of the JACCRO GC-07 study. Neoadjuvant chemotherapy (NAC) is thought to have several advantages over adjuvant settings.

**Objective:**

This study aimed to compare the efficacies of NAC DS and the surgery-first strategy for advanced gastric cancer patients with D2 gastrectomy.

**Methods:**

This was a retrospective, single-institution observational study. Of 171 patients with locally advanced (cStage IIB or III) gastric cancer who underwent curative D2 gastrectomy and received NAC DS and/or S-1 adjuvant chemotherapy between 2011 and 2017, 76 (after propensity score matching for 132 patients who met the eligibility criteria) were enrolled in this study. The 3-year progression-free survival (PFS) rate was used to directly compare efficacies between NAC DS patients and surgery-first patients.

**Results:**

The 3-year PFS rates for the NAC DS group were significantly higher than those for the surgery-first group (80.0 vs. 58.7; *p* = 0.037), and the progression hazard ratio of the NAC DS group compared with the surgery-first group was 0.394 (95% confidence interval 0.159–0.978; *p* = 0.045).

**Conclusions:**

The NAC DS group showed a high 3-year PFS compared with the surgery-first group, with standard S-1 postoperative chemotherapy or observation. NAC DS can be expected to be beneficial as the standard therapy for advanced gastric cancer and should be adopted for the test arm of a randomized controlled phase III trial.

**Electronic supplementary material:**

The online version of this article (10.1245/s10434-019-07299-7) contains supplementary material, which is available to authorized users.

Gastrectomy with D2 lymphadenectomy is a standard surgical procedure for locally advanced gastric cancer in Eastern Asia and is also recommended in Western countries,^1^,^2^ whereas surgery followed by adjuvant chemotherapy for locally advanced gastric cancer is the standard approach in Korea and Japan.^3^–^5^ Surgery plus adjuvant chemotherapy yields a 5-year survival rate of 70%;^6^–^8^ however, patients’ prognosis of a novel therapeutic strategy for locally advanced gastric cancer has been shown to have further reformability than standard D2 gastrectomy and adjuvant chemotherapy.

Neoadjuvant chemotherapy (NAC) has several advantages over adjuvant settings. For instance, it can eradicate systemic micrometastasis^9^,^10^ and achieve downstaging to immediately start systemic treatment. In addition, the start of chemotherapy can be delayed if postoperative complications occur. Furthermore, postoperative compliance of chemotherapy is insufficient for patients who undergo gastrectomy. Indeed, 30–50% of patients drop out of a 1-year course of chemotherapy after surgery.^6^,^7^

S-1 plus cisplatin (SC) is a standard treatment for metastatic gastric cancer in Eastern Asia.^11^ The Japan Clinical Oncology Group (JCOG) is currently conducting a phase III trial of NAC SC followed by surgery, and promising survival results have been reported from a small phase II trial evaluating two courses of NAC SC for bulky nodal disease.^12^ In addition, several phase II studies have demonstrated that NAC SC is safe and feasible.^12^–^14^ However, NAC SC phase III trials for large type 3 or 4 gastric cancers (JCOG0501) have not shown favorable results for NAC for advanced resectable gastric cancer.^15^

In Japan, docetaxel plus S-1 (DS) is thought to be an alternative regimen when the standard SC regimen cannot be used for metastatic gastric cancer.^16^ However, it has been suggested that DS may be superior to S-1 monotherapy with regard to survival in cases with non-measurable lesions.^16^ Paclitaxel and docetaxel are expected to be effective against peritoneal dissemination because of their high affinity for the peritoneum;17 therefore, this combination treatment may be a promising novel therapy for locally advanced gastric cancer.

In the present study, we evaluated the efficacy of NAC with DS for patients with advanced gastric cancer.

## Patients and Methods

We retrospectively investigated 171 patients who underwent curative D2 gastrectomy with or without S-1 adjuvant chemotherapy as the ACTS-GC (Adjuvant Chemotherapy Trial of TS-1 for Gastric Cancer) protocol for cStageIIB or III gastric cancer, between 2011 and 2017. The trial was conducted in accordance with the World Medical Association Declaration of Helsinki and the Japanese Ethical Guidelines for Medical and Health Research Involving Human Subjects. This protocol was approved by the Clinical Research Ethics Committee at Chiba University Hospital.

Patient medical records and survival status were retrospectively reviewed in August 2018. Patients were eligible for this study if they (1) had histologically proven advanced gastric cancer (including large type 3 or 4, or bulky N) with clinical stage IIB or III disease (including T4b [liver, transverse colon, pancreatic body and tail, spleen and diaphragm) after radical gastrectomy with D2 lymph node dissection and R0 surgery; (2) were between 20 and 85 years of age; (3) had received no other treatments; (4) had been treated with S-1 chemotherapy within 8 weeks after surgery if they had (y)pStage II or III gastric cancer; (5) had more than 20 lymph nodes examined; and (6) had no synchronous or metachronous cancer. The data of 132 patients were examined after excluding 39 patients who did not meet these criteria retrospectively.

**Fig. 1:**
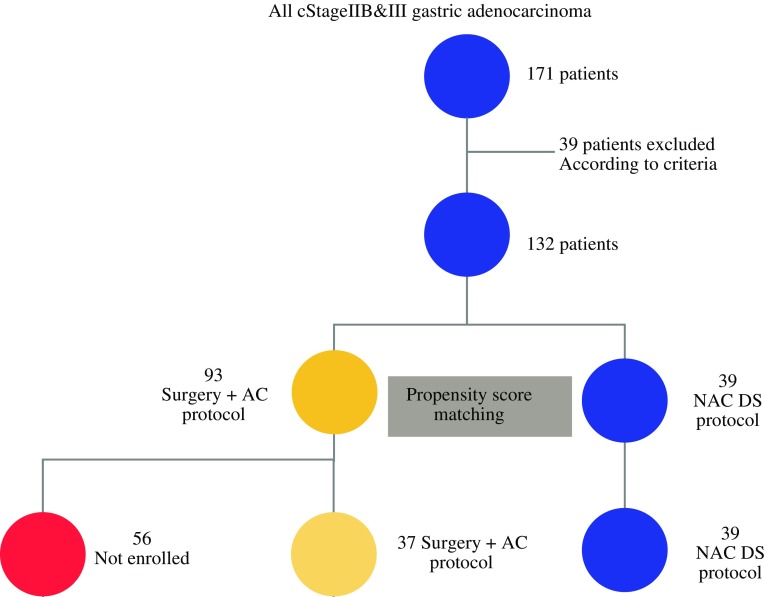
Enrolled patients. Thirty-nine patients were excluded according to the exclusion criteria. All 132 patients remaining after exclusion, along with 76 patients (39 in the NAC DS group and 37 in the surgery-first group) after propensity score matching, were analyzed. *NAC* neoadjuvant chemotherapy, *DS* docetaxel plus S-1

The 3-year progression-free survival (PFS) rate was determined to compare the efficacy of the NAC DS and surgery-first approaches. To reduce selection bias typically caused by the inclusion of imbalanced covariates in retrospective studies, the propensity score-matching method was used (Fig. 1). Tumor stage classification and D classification were in accordance with the Japanese Classification of Gastric Carcinoma (Third English Edition).^18^

### Chemotherapy Regimen

Patients in the NAC DS group received two 21-day courses of NAC with the DS regimen (S-1 80 mg/m^2^ from days 1 to 14, and docetaxel 40 mg/m^2^ on day 1) and then underwent D2 gastrectomy, while patients in the surgery-first group received S-1 adjuvant chemotherapy if they had pStage II or III gastric cancer postoperatively, in accordance with the ACTS-GC protocol.^6^ It was intended that patients in the NAC DS group receive S-1 adjuvant chemotherapy.

### Follow-Up Evaluations

For follow-up clinical studies, the patients’ history and findings on physical examination, chest x-ray, upper gastrointestinal endoscopy, and enhanced computed tomography (eCT) of the abdomen and chest were performed at intervals of 3 months. When necessary, abdominal ultrasonic examinations, positron emission tomography (PET), and general bone scans were additionally examined.

### Statistical Analyses

Clinicopathologic characteristics were summarized to provide a descriptive analysis, and mean ± standard deviations were used to show quantitative variables of the NAC DS and surgery-first groups. Qualitative variables are expressed as frequency and percentage. A two-sample *t*-test for quantitative variables and Chi square test for qualitative variables were used to compare the characteristics of each group.

In non-randomized studies, effect estimates may be biased by group discordance; thus, propensity score-matched analysis was performed to minimize selection bias. Propensity scores were calculated using logistic regression. We included the following baseline covariates: age, sex, body mass index (BMI), Charlson Comorbidity Index, histology, tumor location, cT and cN factors, type of gastric resection, and type of surgical reconstruction. We purposefully used clinical T and N factors and variables collected before initial treatment as these represent the available data that physicians use to make decisions regarding stage prior to treatment. The confounding variables were input into the propensity score model. The nearest-neighbor matching method was applied, and one-to-three matching between groups was achieved. The value of the caliper size was 0.25.

The 3-year PFS rate was the primary endpoint of this study, and was defined as the period from the start of treatment to last follow-up, recurrence date, or death due to any cause, whichever came first. The 3-year PFS rate and 95% confidence interval (CI) were calculated using the Kaplan–Meier method. The PFS rates between the NAC DS and surgery-first groups were compared using the log-rank test. The Cox proportional hazards model was used to calculate the progression hazard ratio (HR) of the NAC DS group, compared with the surgery-first group as the reference, and the 95% CIs. All data analyses were performed by a medical statistician using the SPSS software package for Windows version 24.0 (IBM Corporation, Armonk, NY, USA). All tests were two-sided and statistical significance was set at *p* < 0.05.

### Pathological Response Criteria of Neoadjuvant Chemotherapy (NAC)

Pathological responses for gastric cancer after NAC were evaluated and graded by two pathologists, according to the proportion of necrosis in the tumor: grade 0, no necrosis; grade 1a, less than one-third necrosis; grade 1b, more than one-third or less than two-thirds necrosis; grade 2, less than two-thirds or more than all necrosis; and grade 3, all parts of the tumors affected by necrosis.^19^

## Results

### Patient Characteristics

Of the 132 patients eligible for this study, 39 received NAC DS and 93 received surgery first. The clinicopathologic characteristics of patients in the two groups are summarized in Table 1. Propensity score matching was used to compare the 3-year PFS rates between the two groups; after propensity score matching, 39 patients in the NAC DS group and 37 patients in the surgery-first group were matched. The differences in baseline characteristics between the two groups were statistically significant across the sex covariate before adjusting, however these differences were all eliminated without significant differences between the two groups after adjusting with propensity score matching (Table 1).

**Table 1:** Demographics and clinicopathological characteristics in propensity score matched patients

Factors	All patients			Matched patients		
	NAC	Surgery first	*P*	NAC	Surgery first	*P*
	(*N* = 39)	(*N* = 93)		(*N* = 39)	(*N* = 37)	
Age	69.3 ± 7.76	70.2 ± 10.9	0.378	69.3 ± 7.76	70.4 ± 8.50	0.557
*Sex*, *n* (%)						
Male	32 (82.1)	59 (62.8)	0.0207	32 (82.1)	29 (78.4)	0.777
Female	7 (17.9)	37 (39.4)		7 (17.9)	8 (21.6)	
BMI	22.4 ± 3.12	22.4 ± 3.44	0.922	22.4 ± 3.12	22.2 ± 3.13	0.811
*Charlson comorbidity index*						
0	21 (53.8)	50 (53.2)	0.664	21 (53.8)	20 (54.1)	0.756
1	11 (28.2)	20 (21.3)		11 (28.2)	10 (27.0)	
2	5 (12.8)	14 (14.9)		5 (12.8)	3 (8.1)	
3	2 (5.1)	10 (10.6)		2 (5.1)	4 (10.8)	
*Histology*, *n* (%)						
Intestinal	10 (25.6)	12 (12.8)	0.157	10 (25.6)	8 (21.6)	0.345
Diffuse	7 (17.9)	25 (26.6)		7 (17.9)	12 (32.4)	
Mixed	22 (56.4)	57 (60.6)		22 (56.4)	22 (59.5)	
*Location of the tumor*						
U	15 (38.5)	25 (26.6)	0.330	15 (38.5)	12 (32.4)	0.511
M	9 (23.1)	31 (33.0)		9 (23.1)	13 (35.1)	
L	15 (38.5)	38 (40.4)		15 (38.5)	12 (32.4)	
*cT category*, *n* (%)						
cT1 (M/SM)	1 (2.6)	0 (0)	0.158	1 (2.6)	0 (0)	0.471
cT2 (MP)	1 (2.6)	9 (9.6)		1 (2.6)	3 (8.1)	
cT3 (SS)	20 (51.3)	56 (59.6)		20 (51.3)	19 (51.4)	
cT4a (SE)	16 (41.0)	25 (26.6)		16 (41.0)	12 (32.4)	
cT4b (SI)	1 (2.6)	4 (4.3)		1 (2.6)	3 (8.1)	
*cN category*, *n* (%)						
N0	5 (12.8)	6 (3.6)	0.220	5 (12.8)	2 (5.4)	0.264
N1–3	34 (87.2)	88 (92.8)		34 (87.2)	35 (94.6)	
*cStage*, *n* (%)						
cIIB	17 (43.6)	27 (28.7)	0.0804	17 (43.6)	10 (27.0)	0.1316
cIIIA-C	22 (56.4)	67 (71.3)		22 (56.4)	27 (73.0)	
*Radiographic type 4 or Large* (≥ 8 cm) *type 3*						
Yes	11 (28.2%)	24 (25.8%)	0.7758	11 (28.2%)	10 (27.2%)	0.909
No	28 (71.8%)	69 (74.2%)		28 (71.8%)	27 (72.8 %)	
*Gastric resection*						
Distal Gastrectomy	16 (41.0)	46 (48.9)	0.405	16 (41.0)	15 (40.5)	0.966
Total gastrectomy	23 (59.0)	48 (51.1)		23 (59.0)	22 (59.5)	
*Reconstruction*						
Billroth I	5 (12.8)	14 (14.9)	0.214	5 (12.8)	6 (16.2)	0.485
Billroth II	1 (2.6)	11 (11.7)		1 (2.6)	3 (2.6)	
Roux-en-Y	33 (84.6)	69 (73.4)		33 (84.6)	28 (75.7)	

*M* mucosa/muscularis mucosa, *SM* submucosa, *MP* muscularis propria, *SS* subserosa, *SE* serosa, *SI* invasion of adjacent structures

### Surgery and Postoperative Complications

A total of 39 patients underwent D2 gastrectomy after NAC. The median operation time was 311 min (range 99–590), which was significantly longer than in the surgery-first group (*p* = 0.006, Mann-Whitney U-test), and the estimated blood loss was 645 g (range 5–2950), which was not markedly different from that in the surgery-first group (*p* = 0.086) after propensity score matching (Table 2).

**Table 2:** Postoperative complications according to the Clavien–Dindo classification

Factors	All patients			Matched patients		
	NAC	Surgery first	*P*	NAC	Surgery first	*P*
	(*N* = 39)	(*N* = 94)		(*N* = 39)	(*N* = 37)	
*Operative time (min)*						
Median (range)	311 (99–590)	279 (124–575)	0.001	311 (99–590)	265 (124–519)	0.006
*Intraoperative bleeding* (g)						
Median (range)	645 (5–2950)	526 (35–3700)	0.001	645 (5–2950)	380 (100–3700)	0.086
*Postoperative complications*						
Total (%)	9 (23.1)	38 (40.4)	0.0567	9 (23.1)	15 (40.5)	0.101
Grade 1	4 (10.3)	17 (18.1)	0.260	4 (10.3)	2 (5.4)	0.433
Grade 2	2 (5.1)	11 (11.7)	0.245	2 (5.1)	7 (18.9)	0.063
Grade 3	3 (7.7)	7 (7.4)	0.961	3 (7.7)	6 (16.2)	0.250
Grade 4	0 (0.0)	4 (4.3)	0.191	0 (0.0)	1 (2.8)	0.301
*Complications of all grade*, (%)						
Anastomotic leakage	1 (2.6)	1 (1.1)	0.518	1 (2.6)	1 (2.7)	0.970
Pancreatic fistula	6 (15.4)	9 (9.6)	0.335	5 (15.4)	5 (13.5)	0.929
Anastomotic stricture	0 (0.0)	3 (3.2)	0.259	0 (0.0)	0 (0.0)	
Bowel obstruction	0 (0.0)	5 (5.3)	0.142	0 (0.0)	2 (5.4)	0.141
Intraabodominal abscess	0 (0.0)	2 (2.1)	0.359	0 (0.0)	1 (2.7)	0.301
Pneumonia	0 (0.0)	6 (6.4)	0.106	0 (0.0)	3 (8.1)	0.067
Cholecystitis	0 (0.0)	3 (3.2)	0.259	0 (0.0)	0 (0.0)	1.000
Others	2 (5.1)	10 (10.6)	0.313	2 (5.1)	1 (2.7)	0.587
*Complications of grade* 3–4						
Pancreatic fistula	3 (7.7)	6 (6.4)	0.784	3 (7.7)	2 (5.4)	0.643
Anastomotic stricture	0 (0.0)	1 (1.1)	0.518	0 (0.0)	0 (0.0)	1.000
Bowel obstruction	0 (0.0)	2 (2.1)	0.364	0 (0.0)	1 (2.7)	0.301
Pneumonia	0 (0.0)	2 (2.1)	0.364	0 (0.0)	0 (0.0)	1.000
*Postoperative hospital stay* (*days)*						
Median (range)	14 (9–57)	15 (7–106)	0.397	14 (9–57)	14 (10–70)	0.695

Gr, Grade; Clavien–Dindo Classification

^a^The total does not add up because of overlapping elements

Postoperative complications were classified according to the Clavien–Dindo classification (Table 2).^20^ Complications of any grade in the NAC DS group occurred in nine patients (23.1%), and the highest grade of complications was grade 3a in three patients (7.7%). No patients died postoperatively. The rates of postoperative complications in the NAC DS group did not differ significantly from those in the surgery-first group after propensity score matching (Table 2).

### Pathological Findings and Downstaging after NAC

Two patients achieved a pathological complete response after NAC DS (5.1%). A pathological response of grade 0, 1a, 1b, and 2 was observed in 5 (12.8%), 12 (30.8%), 9 (23.1%), and 11 (28.2%) patients, respectively. Data on the distribution of baseline cT, cN and cStage (Table 1), and (y)pT, (y)pN and (y)pStage (Table 3) are shown. Although the baseline cT, cN, and cStage values were not significantly different between the two groups, the rate of ypT in the NAC DS group was significantly lower than the rate of pT in the surgery-first group.

**Table 3:** Pathological findings in resected patients

Factors	All patients			Matched patients		
	NAC	Surgery first	*P*	NAC	Surgery first	*P*
	(*N* = 39)	(*N* = 94)		(*N* = 39)	(*N* = 37)	
*pT category*, *n* (%)						
pT0	2 (5.1)	0 (0)	0.0002	2(5.1)	0 (0)	0.027
pT1(M/SM)	5 (12.9)	7 (7.4)		5(12.9)	1 (2.7)	
pT2(MP)	12 (30.8)	7 (7.4)		12 (30.8)	5 (13.5)	
pT3(SS)	13 (33.3)	32 (34.0)		13 (33.3)	12 (32.4)	
pT4a(SE)	7 (17.9)	39 (41.5)		7 (17.9)	12 (32.4)	
pT4b(SI)	0 (0)	9 (9.6)		0 (0)	4 (10.8)	
*pN category*, *n* (%)						
pN0	15 (38.5)	13 (13.8)	0.0126	15 (38.5)	6 (16.2)	0.1466
pN1	8 (20.5)	25 (26.6)		8 (20.5)	11 (29.7)	
pN2	9 (23.1)	31 (33.0)		9 (23.1)	10 (27.0)	
pN3	6 (15.4)	25 (26.6)		6 (15.4)	10 (27.0)	
*pStage*, *n* (%)						
pT0N0	2 (5.1)	0 (0.0)	0.001	2 (5.1)	0 (0.0)	0.0966
pIA-B	10 (25.6)	8 (8.5)		10 (25.6)	6 (16.2)	
pIIA-B	13 (33.3)	23 (24.5)		13 (33.3)	8 (21.6)	
pIIIA-C	14 (35.9)	63 (67.0)		14 (35.9)	23 (62.2)	

### Progression-Free Survival

**Fig. 2:**
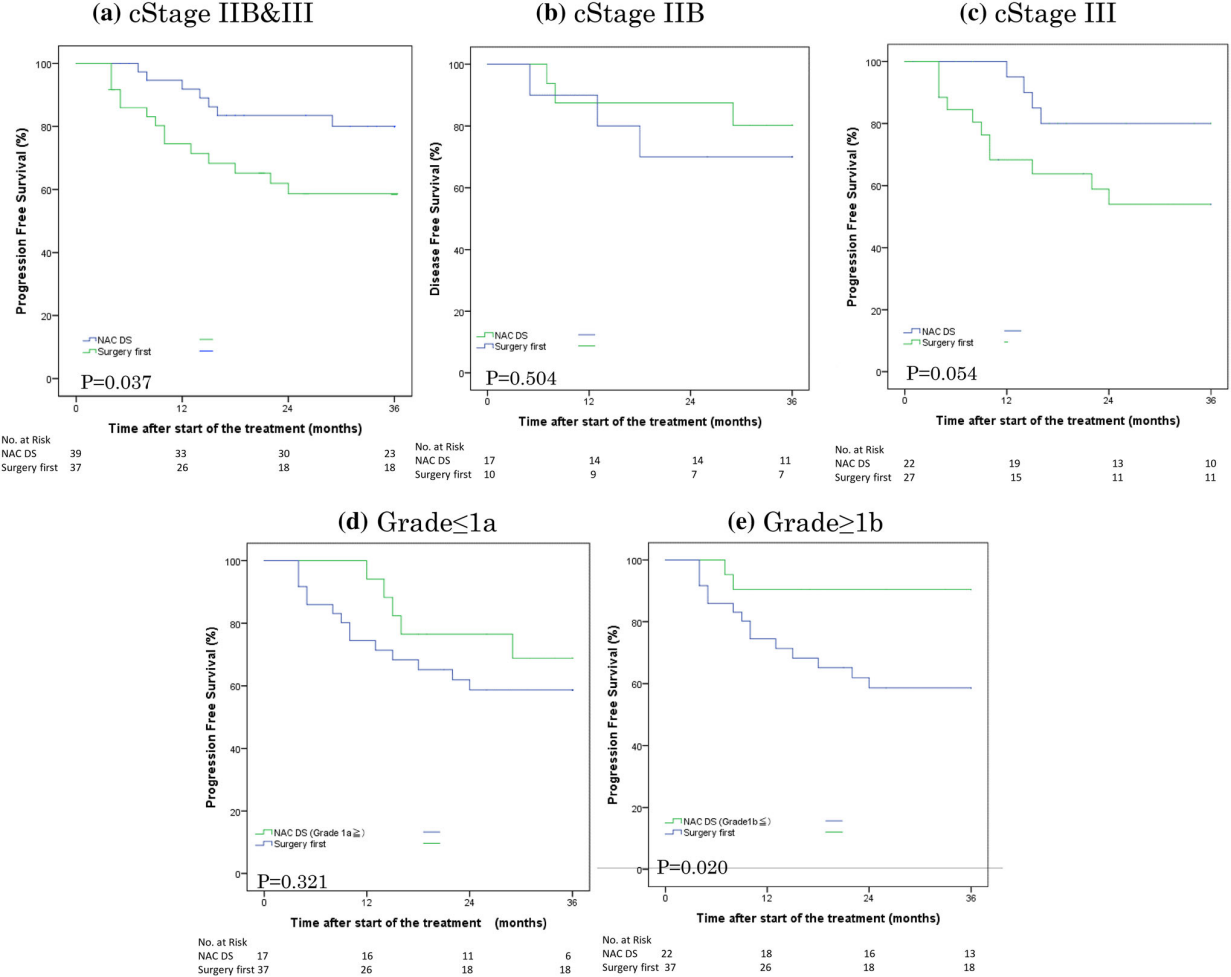
(**a**–**c**) Progression-free survival of patients with cStage IIB and III, cStage IIB, and cStage III gastric cancer using the Kaplan–Meier method. (**d**, **e**) Progression-free survival of patients with grade ≤ 1a and grade ≥ 1b pathological responses after NAC DS compared with the surgery-first group using the Kaplan–Meier method. *NAC* neoadjuvant chemotherapy, *DS* docetaxel plus S-1

The 3-year PFS rates were compared between the NAC DS and surgery-first groups (Table 4). The PFS rate in the NAC DS group was 80.0% (95% CI 67.1–93.4%), which was significantly higher (*p* = 0.037) than in the surgery-first group (58.7%, 95% CI 41.9–75.4%). A comparison between the NAC DS and surgery-first groups according to cStage (IIB or III) showed no marked differences between the two groups. For cStage IIB gastric cancer, the PFS rate for the NAC DS group (80.2%, 95% CI 60.0–100.4%) did not differ significantly from that in the surgery-first group (70.0%, 95% CI 41.6–98.4%; *p* = 0.504). Furthermore, no marked differences in the PFS rates for cStage III (IIIA, IIIB, and IIIC) patients were noted between the NAC DS (80.0%, 95% CI 62.5–97.5%) and surgery-first groups (54.0%, 95% CI 33.6–74.4; *p* = 0.054). The PFS graphs of patients with cStage IIB and/or III gastric cancer are shown in Fig. 2a.

**Table 4:** Disease-free survival rate for the NAC DS and Surgery first groups according to stages (Japanese Classification of Gastric Carcinoma (Third English Edition) using the log-rank test

Stages	NAC DS	Surgery first	*p* value
	(*n* = 39)	(*n* = 37)	
All	80.0 (67.1–93.4)	58.7 (41.9–75.4)	0.037
cStage IIB	80.2 (60.0–100.4)	70.0 (41.6–98.4)	0.504
cStage III	80.0 (62.5–97.5)	54.0 (33.6–74.4)	0.054

Pathological response	NAC DS	Surgery first	*p* value
	(*n *= 39)	(*n* = 37)	
Grade 1a ≧	76.4 (56.3–96.6)	58.7 (41.9–75.4)	0.321
Grade 1b ≦	90.5 (77.9–103.0)		0.020

A comparison of the NAC DS and surgery-first groups according to postoperative pathological responses (grade ≤ 1a or ≥ 1b) showed differences in grade ≥ 1b responses between the two groups. The PFS rate for cases with a grade ≤ 1a pathological response after NAC DS and surgery (76.4%, 95% CI 56.3–96.3%) did not differ significantly from that in the surgery-first group (*p* = 0.321). Additionally, the PFS rate for cases with a grade ≥ 1b pathological response after NAC DS and surgery (90.5%, 95% CI 77.9–103.0) was higher than in the surgery-first group (*p* = 0.020). The PFS graphs of patients with grade ≤ 1a or ≥ 1b pathological responses after NAC DS compared with the surgery-first group are shown in Fig. 2b.

### A Comparison of the Hazard Ratios for Recurrence Between the NAC DS and Surgery-First Groups

The HR for progression of the NAC DS group was calculated using the Cox proportional hazard model to compare surgery first as the reference (Table 5). The HR of the NAC DS group for all patients after propensity score matching was 0.394 (95% CI 0.159–0.978; *p* = 0.045). This study could not show that NAC DS was significantly related to a decreased risk of recurrence for patients with stage IIB (HR 0.583, 95% CI 0.118–2.892; *p* = 0.509) or stage III disease (HR 0.344. 95% CI 0.109–1.083; *p* = 0.068). The HR of gastric cancer progression in cases with grade ≥ 1b postoperative pathological responses compared with surgery first as the reference was 0.207 (95% CI 0.047–0.912; *p* = 0.037). Furthermore, this study did not find NAC DS to be significantly associated with a decreased risk of gastric cancer recurrence for patients with grade ≥ 1a pathological responses (HR 0.602, 95% CI 0.216–1.679; *p* = 0.330).

**Table 5:** Hazard ratio of NAC DS for recurrence of gastric cancer compared with surgery first as a reference

Stages	Hazard ratio (95% CI)	*p* value
All	0.394 (0.159–0.978)	0.045
cStage IIB	0.583 (0.118–2.892)	0.509
cStage III	0.344 (0.109–1.083)	0.068
Grade 1a ≧	0.602 (0.216–1.672)	0.330
Grade 1b ≦	0.207 (0.047–0.912)	0.037

## Discussion

The effectiveness of DS adjuvant chemotherapy for pStage III gastric cancer was recently reported (JACCRO GC-07) at the 2018 American Society of Clinical Oncology annual meeting.^5^ The DS protocol of this study, in which its safety and efficacy as adjuvant chemotherapy for pStage III gastric cancer in a phase III RCT (JACCRO GC-07) was proven.

We completed a phase II trial for evaluation of the safety of NAC DS for patients with locally advanced gastric cancer followed by standard D2 gastrectomy. In the phase II trial (in submission), the authors judged NAC DS and surgery to be acceptable for daily clinical use based on the completion rate of NAC and surgery, R0 resection rate, adverse events of chemotherapy, and surgery. Staging laparoscopy was required in all cases prior to NAC treatment for advanced gastric cancer patients, to obtain a precise diagnosis of cancer staging.

We selected clinical stage IIB and III gastric cancer patients for this study because the 5-year survival rate for clinical stage IIB gastric cancer is < 70%, therefore a more effective treatment than D2 gastrectomy with adjuvant chemotherapy is needed in this field.

Our present study showed that the 3-year PFS rates in the NAC DS group for cStage IIB and III gastric cancer patients were higher than those in the surgery-first group. Furthermore, the HR of progression in the NAC DS group, compared with the surgery-first group, was 0.394.

This study had some limitations as it was a retrospective study and only early outcomes were observed. Although the 3-year PFS is meaningful because most gastric cancer recurrences are known to occur within 3 years,^21^ an expanded or well-designed prospective RCT is warranted in order to confirm our results.

Nevertheless, the present results suggest that NAC DS chemotherapy should be considered for the test arm of an RCT, compared with standard treatment. Only patients from a single institution were enrolled in this study; therefore, an international study with standardized D2 dissection may lead to more valuable research results.

Whether doublet or triplet regimens should be used for NAC is controversial. Satisfactory 3-year overall survival rates of perioperative triplet regimens in a phase II trial have been reported,^22^–^24^ and reports on perioperative chemotherapy using triplet regimens have shown a high rate of grade 3/4 adverse events. Furthermore, phase III trials of docetaxel and cisplatin combination therapy for metastatic gastric cancer (JCOG1013) have not shown favorable results.^25^

Relatively large differences among pathological responses in the 3-year PFS and HR of the risk of gastric cancer progression were observed in this study, suggesting that the development of a companion diagnosis for NAC is important.

Treatment approaches using molecular-targeted drugs and immune checkpoint inhibitors for metastatic gastric cancer treatment are currently being developed,^26^–^29^ and are promising from the point of view of precision oncology.^30^–^32^ In the nearest future, molecular-targeted drugs and immune checkpoint inhibitors will appear in the field of perioperative chemotherapy development.

## Conclusions

NAC DS with D2 gastrectomy had a higher PFS rate than the surgery-first approach for patients with cStage IIB and III gastric cancer. In addition, surgery first was found to be associated with more than twice the risk of progression as NAC DS. Although previous studies have not confirmed the effectiveness of NAC for advanced gastric cancer, NAC DS should be considered preferentially for the test arm of an RCT for patients with locally advanced gastric cancer.

## Electronic supplementary material

Below is the link to the electronic supplementary material.

Supplementary material 1 (PDF 38 kb)

Supplementary material 2 (PDF 46 kb)

Supplementary material 3 (PDF 45 kb)
