# Supplementary material for: Survival Benefit of Neoadjuvant Chemotherapy with S-1 Plus Docetaxel for Locally Advanced Gastric Cancer: A Propensity Score-Matched Analysis
Source: Ann Surg Oncol. 2019 Apr 11;26(6):1805–13. doi: 10.1245/s10434-019-07299-7 (PMC6510880; doi:10.1245/s10434-019-07299-7)
Supplement: Supplementary file 1 — Supplementary material 1 (PDF 38 kb) [file 10434_2019_7299_MOESM1_ESM.pdf]

Supple. TABLE 1. Adverse Events Associated with Neoadjuvant Chemotherapy

(NAC) According to the NCI-CTC Grade

	(Number of patients)				
Adverse Event	Gr 1	Gr 2	Gr 3	Gr 4	Gr 3/4(%)
Hematologic					
Leukocytopenia	9	8	9	0	23.1
Neutrocytopenia	0	4	9	1	25.6
Anemia	0	2	2	0	5.1
Thrombocytopenia	1	0	0	0	0.0
Total bilirubin	0	0	1	0	2.6
AST	0	0	2	0	5.1
ALT	0	0	2	0	5.1
Non-hematologic					
Nausea	3	0	2	0	5.1
Vomiting	1	0	0	0	0.0
Diarrhea	0	4	3	0	7.7

Appetite loss	1	0	2	0	5.1
General malaise	1	0	0	0	0.0
Skin reaction	1	2	0	0	0.0
Alopecia	1	0	0	0	0.0
Febrile neutropenia	0	0	1	0	2.6
Epistaxis	1	0	0	0	0.0

Gr, Grade; Common Terminology Criteria for Adverse Events (CTCAE) v4.0
