# Supplementary material for: Survival Benefit of Neoadjuvant Chemotherapy with S-1 Plus Docetaxel for Locally Advanced Gastric Cancer: A Propensity Score-Matched Analysis
Source: Ann Surg Oncol. 2019 Apr 11;26(6):1805–13. doi: 10.1245/s10434-019-07299-7 (PMC6510880; doi:10.1245/s10434-019-07299-7)
Supplement: Supplementary file 2 — Supplementary material 2 (PDF 46 kb) [file 10434_2019_7299_MOESM2_ESM.pdf]

Supple. TABLE 2. Postoperative S-1 administration after for the NAC DS and Surgery first groups according to stages (Japanese Classification of Gastric Carcinoma (Third English Edition)

(y)pStages	NAC DS (n=39)	surgery first (n=37)	p-value
Cases with postoperative S-1 administration for 1 year or under			
All (yes/no)	33 (84.6%)/ 6	24 (64.9%)/ 13	p=0.0469
Excluding pStage I for upfront surgery	33 (84.6%)/ 6	24 (77.4%)/ 7	p=0.4419
ypT0N0 (yes/no)	1 (50.0%)/ 1	0/ 0	
(y)pStageIA-B (yes/no)	9 (90%)/ 1	0 (0.0%)/ 6	

(y)pStageIIA-B (yes/no)	11 (84.6%)/ 2	6 (75%)/ 2	p=0.5858
(y)pStageIIIA-C (yes/no)	12 (85.7%)/ 2	18 (78.3%) / 5	p=0.5745

Complete Postoperative S-1 administration cases for 1 year

All (yes/no)	15 (38.5%)/ 24	18 (48.6%)/ 19	p=0.3705
Excluding pStage I for upfront surgery	15 (38.5%)/ 24	18 (58.1%)/ 13	p=0.1027
ypT0N0 (yes/no)	0 (0.0%)/ 2	0/ 0	
(y)pStageIA-B (yes/no)	4 (40%)/ 6	0 (0.0%)/ 6	
(y)pStageIIA-B (yes/no)	3 (23.1%)/ 10	6 (75%)/ 2	p=0.0195
(y)pStageIIIA-C (yes/no)	8 (57.1%)/ 6	12 (52.2%) / 11	p=0.7687
