# Supplementary material for: Survival Benefit of Neoadjuvant Chemotherapy with S-1 Plus Docetaxel for Locally Advanced Gastric Cancer: A Propensity Score-Matched Analysis
Source: Ann Surg Oncol. 2019 Apr 11;26(6):1805–13. doi: 10.1245/s10434-019-07299-7 (PMC6510880; doi:10.1245/s10434-019-07299-7)
Supplement: Supplementary file 3 — Supplementary material 3 (PDF 45 kb) [file 10434_2019_7299_MOESM3_ESM.pdf]

Supple. TABLE 3. Postoperative S-1 administration after for the NAC DS groups according to pathological responses (Japanese Classification of Gastric Carcinoma (Third English Edition)

Pathological grade	NAC DS (n=39)
Cases with postoperative S-1 administration for 1 year or under	
All (yes/no)	33 (84.6%)/ 6
Grade 0 (yes/no)	4 (80%)/ 1
Grade1a (yes/no)	10 (83.3%)/ 2
Grade 1b (yes/no)	8 (88.9%)/ 1
Grade 2 (yes/no)	10 (90.9%)/ 1
Grade 3 (yes/no)	1 (50%)/ 1
Cases with postoperative S-1 administration for 1 year	
All (yes/no)	15 (38.5%)/ 24

Grade 0 (yes/no)	2 (40%)/ 3
Grade1a (yes/no)	6 (50%)/ 6
Grade 1b (yes/no)	3 (33.3%)/ 6
Grade 2 (yes/no)	4 (36.4%)/ 7
Grade 3 (yes/no)	0 (0.0%)/ 2
